# Evaluating the Effect of Nursing-Performed Point-of-Care Ultrasound on Septic Emergency Department Patients

**DOI:** 10.7759/cureus.40519

**Published:** 2023-06-16

**Authors:** Sharmin Kalam, Nicholas Selden, Korbin Haycock, Tammy Lowe, Heather Skaggs, Vi Am Dinh

**Affiliations:** 1 Emergency Medicine, Loma Linda University Medical Center, Loma Linda, USA; 2 Emergency Medicine, Sutter Medical Center, Sacramento, USA; 3 Emergency Medicine, Riverside University Health System Medical Center, Moreno Valley, USA; 4 Nursing, Riverside University Health System Medical Center, Moreno Valley, USA; 5 Internal Medicine, Division of Pulmonary and Critical Care, Loma Linda University Medical Center, Loma Linda, USA

**Keywords:** management, diagnosis, sepsis, np-pocus, pocus, ultrasound

## Abstract

Introduction

Nursing-performed point-of-care ultrasound (NP-POCUS) studies have been performed on applications such as ultrasound-guided peripheral intravenous line placement and assessing bladder volume. However, research on the use of NP-POCUS in the management of septic patients remains limited. The purpose of this quality improvement study was to investigate how NP-POCUS could impact fluid treatment decisions affecting septic patients in the emergency department (ED) using a focused IVC and lung ultrasound protocol.

Methods

Nurses received standardized training in POCUS and performed inferior vena cava (IVC) and lung ultrasound scans on septic patients in the ED at predetermined intervals (hours: zero, three, and six). Based on their findings, they were asked to make recommendations on fluid management. Emergency physicians (EPs), both residents and attendings, are providing recommendations for fluid management without the use of ultrasound, which is being compared to the nurse-driven POCUS assessment of fluid management. EPs reviewed the NP-POCUS assessments of patient fluid status to determine nursing accuracy.

Results

A total of 104 patients were scanned, with a mean age of 60.7 years. EPs agreed with nursing ultrasound assessments in 99.1% of cases. Nursing ultrasound images changed management or increased physician confidence in current treatment plans 83.7% and 96.6% of the time, respectively. Before reviewing saved nursing ultrasound images, EPs underestimated fluid tolerance in 37.5% of cases, overestimated fluid tolerance in 26% of cases, and correctly estimated fluid tolerance (within 500 ml) in 36.5% of cases. Throughout resuscitation, IVCs became less collapsible, the number of cases with B-lines was essentially unchanged, and less fluid was recommended.

Conclusion

This study demonstrated that nurse-performed POCUS is feasible and may have a meaningful impact on how physicians manage septic patients in the emergency department.

## Introduction

Ultrasound (US) imaging has proven to be an invaluable diagnostic tool for modern physicians, and the application of this technology has evolved tremendously since its invention in the 1940s [1-6]. Point-of-care ultrasound (POCUS) is a modern technique defined as bedside US imaging performed directly by a healthcare professional. In the emergency department (ED), POCUS has emerged as a valuable tool for rapid diagnosis, evaluation, and even procedures for patients seeking emergent care [2,6]. This diagnostic modality provides real-time US images that are used by medical providers to improve diagnostic accuracy, and POCUS has also been shown to help physicians make treatment decisions leading to better patient outcomes [7-10]. While POCUS has been primarily used by physicians, a growing movement to train nurses in the use of POCUS has prompted researchers to investigate its benefits in patient management and discover new applications for patient care.

To date, several studies have demonstrated the benefit of NP-POCUS in guiding intravenous (IV) line access and, to a lesser extent, in urinary catheterization and assessing for heart failure [11-13]. When applied to IV access procedures, POCUS training reduced the number of attempts needed to successfully complete the procedure and led to greater patient satisfaction [14-18]. Similar results were observed for urinary catheterizations when nurses utilized pre-procedure POCUS to more accurately measure bladder volume and also when scanning for pleural effusions caused by heart failure [11,12].

Research also shows nurses are capable of using POCUS to accurately measure the inferior vena cava (IVC), a technique proven to be a reliable and non-invasive way to estimate a patient’s intravascular fluid status [13,19-21]. The lungs can also be used to evaluate for the presence of interstitial edema and possible fluid overload by looking for the ultrasonographic finding known as “B-lines” [22]. There have been no studies to date examining the effects of nursing performed focused IVC and lung ultrasound on the management of septic patients for fluid responsiveness. We focused on the nurses since they are primarily caring for patients at the bedside and are therefore more suitable for serial fluid assessments and may recommend resuscitative interventions that can be continued inpatient. The primary purpose of this quality improvement study was to understand how the treatment decisions affecting septic patients in the emergency department could be impacted by NP-POCUS using a focused IVC and lung ultrasound protocol. We hypothesize that NP-POCUS can reliably determine appropriate fluid assessment in septic patients.

## Materials and methods

The study was a quality improvement (QI) project modeled after a prospective observational design. The study was conducted at a single county teaching hospital ED, which serves over 100,000 annual visits and hosts an emergency medicine residency program. The period during which the sample was collected was from August 2016 to November 2016. Inclusion criteria include patients at least 18 years of age and screened positive per vital signs and presenting complaint, confirmed severe sepsis or septic shock, or any combination of the above. Criteria for confirming severe sepsis included a suspected or known infection, two or more systemic inflammatory response syndrome (SIRS) criteria, and organ dysfunction. Septic shock criteria were defined as refractory hypotension and/or lactate greater than or equal to four after adequate fluid resuscitation [23]. Adequate fluid resuscitation was defined as either receiving 30 ml/kg of fluids or as determined by IVC collapsibility and/or delta stroke volume of < 10% with a straight leg raise or fluid challenge for mechanically ventilated patients and/or stroke volume variation of < 13% [24,25]. Exclusion criteria include patients who were transferred from another hospital with the diagnosis of sepsis. The local Institutional Review Board approved the study as a quality improvement (QI) project.

A pre-existing specialized team of 10 nurses (the Code Team) responded to all in-hospital emergencies such as stroke, cardiopulmonary arrest, rapid response, pulmonary embolism, and, in our case, sepsis, including in the ED. The nurses all had at least three years of critical care clinical practice in an ED or ICU. They received advanced training in hemodynamic assessments with a minimum of 90 days of mentorship by trained preceptors, along with training in the management of sepsis, stroke, and cardiopulmonary arrest resuscitation. Before this study, the Code Team nurses were performing other hemodynamic modalities for septic patients, such as passive straight leg raise and evaluating stroke volume and stroke volume variation using Flotrac via arterial line. They are a stand-alone team that follows sepsis patients at specific intervals as needed for up to 24 hours to ensure hospital bundle measures are met and clinical improvement of patients' status. 

For this study, the code team completed the ultrasound training directed by a US-trained faculty member and the medical director to perform fluid assessment looking at IVC and lung ultrasound for septic patients in the ED. The code team nurses completed three online SonoSim modules (SonoSim, Inc., Santa Monica, CA; fundamentals, IVC/Aorta, pulmonary; four hours total) followed by three one-hour live simulation sessions taught by physicians with healthy volunteers. A nurse competency checklist was developed, and nurses were considered competent after successfully completing 10 sessions. This competency is updated annually to reflect new modalities and is certified by the Medical Director. The machine used in the US was the SPARQ bedside by Philips (Philips Healthcare, Andover, MA).

**Figure 1:**
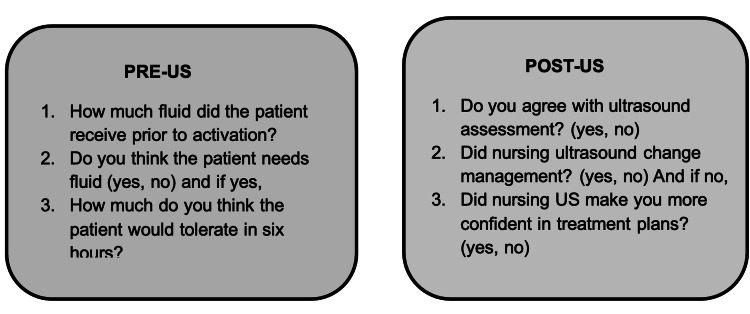
Pre- and post-ultrasound assessment surveys for physicians US = Ultrasound

A study source document was developed in Qualtrics (Qualtrics, LLC, Provo, UT) to collect data from EPs (Figure 1). Upon the patient being triaged, if they met sepsis criteria due to SIRS criteria with a suspected or confirmed source of infection, the chart was activated for sepsis, which is our time zero. The code team would then respond to this sepsis alert within the hour and perform their assessment. The diagnosis was confirmed by the sepsis coordinator and reviewed by both the sepsis committee and the performance improvement and safety committee. Once a septic patient was identified, EPs were asked questions regarding the patient's fluid status and total fluid requirements for the first six hours in the ED. EPs were blinded before NP-POCUS assessments until after they made their initial recommendations for fluid management. EPs then reviewed NP-POCUS assessments and answered additional three questions. These later three questions were repeated at hours three and six. Patient demographic information was noted (including date of birth, age, and gender), as well as data such as vitals (temperature, heart rate, respiratory rate, MAP, oxygen saturation) and lab values for WBC, lactate, creatinine, blood urea nitrogen, and total bilirubin. The present complaint was categorized as respiratory, urinary, abdominal, skin, altered mental status, or other. All administered fluids were measured in liters; the amount given was decided by the physician to assess fluid tolerance. Per the Surviving Sepsis Campaign, the goal was to resuscitate within six hours, and 30cc/kg was used during the study.

**Figure 2:**
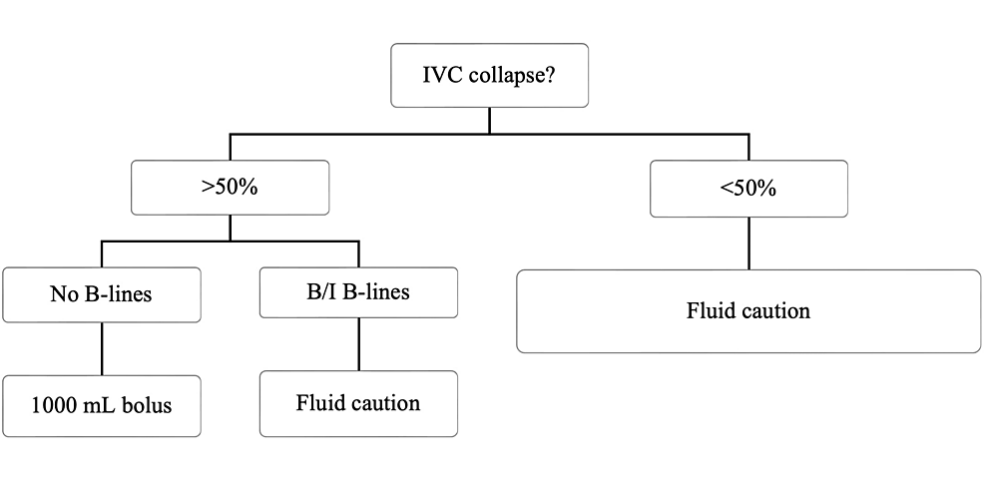
Algorithm for fluid administration in hypotensive patients based on nursing-performed ultrasound. IVC = Inferior vena cava, I B-lines = indeterminate B-lines. Data analysis was calculated using percentage calculations on Microsoft Excel (version 14.2; Microsoft, Redmond, WA).

The NP-POCUS assessment was done within the first hour, hour three, and before hour six, as well as when any additional fluid boluses were needed. POCUS did not add time to the initial fluid bolus since this was all done independently of the physician ordering the fluid bolus. Nurses assessed the inferior vena cava (IVC) for collapsibility greater than 50%. Lung fields were scanned anteriorly and at the mid-axillary line for the presence of B-lines or effusions. Nursing staff made fluid resuscitation recommendations to EPs based on US findings and a resuscitation algorithm (Figure 2). US images were saved and presented to EPs for evaluation and survey completion. The EPs were composed of EM-trained residents and board-certified attendings who have hospital privileges to perform POCUS per the institutional credentialing committee, with an annual review of cases to meet continuation of credentialing. The EPs can independently administer resuscitative fluids upon their clinical assessments and may have utilized POCUS for their assessment; however, that was not tracked for the study. Total fluid resuscitation volume at six hours was recorded. 

Qualitative data was collected when a survey asked EPs about the NP-POCUS images and their interpretations at predetermined intervals (hours: zero, three, and six). The survey asked if the physicians agreed with the nurses’ interpretation if ultrasound changed physician management and if ultrasound increased physician confidence in the treatment plan. The survey also assessed how well the EPs estimated fluid tolerance in the first six hours independently before NP-POCUS evaluation. Based on the gathered data from the survey, percentage calculations were performed using Microsoft Excel (version 14.2; Microsoft, Redmond, WA).

## Results

Ten nurses comprising the Code Team completed the online and hands-on training to achieve the competency required to perform the US procedures. Table 1 provides demographic details regarding the nursing group. 

**Table 1:** Code team nurse demographics (n=10). BSN: Bachelor of Science in Nursing, ADN: Associate's Degree in Nursing, ICU: Intensive Care Unit, EM: Emergency Medicine.

Gender	n (%)
Male	4 (40.0)
Female	6 (60.0)
Experience	4 to 20 years (mean = 10.6)
Highest nursing degree	
BSN	4 (40.0)
ADN	6 (60.0)
Specialty	
ICU*	6 (60.0)
EM	4 (40.0)

The team enrolled 104 septic patients with a mean age of 60.7 years. Males represented almost 50% of the subjects, and the top three chief complaints were abdominal (29.8%), respiratory (25.0%), and altered mental status (22.1%). Table 2 lists the initial vital signs and laboratory values. 

**Table 2:** Patient demographics (n=104) SD: Standard Deviation, EMR: Electronic Medical Record, WBC: White Blood Cell. They all met severe sepsis or septic shock criteria.

Patient demographics	
Mean age, years ± SD	60.7 ± 18.4
Male sex (%)	51 (49.5)
Presenting chief complaint from EMR, n (%)	
Respiratory	26 (25.0)
Urinary	10 (9.6)
Abdominal	31 (29.8)
Skin	1 (1)
Altered mental status	23 (22.1)
Other	13 (12.5)
Mean initial vital signs ± SD	
Temperature (Celsius)	37.4 ± 1.4
Heart rate	110.3 ± 21.7
Mean arterial pressure, mm Hg	84.5 ± 22.9
Respiratory rate	23.7 ± 9.9
Pulse oximetry, SpO2	95.4 ± 4.4
Mean initial laboratory values ± SD	
WBC	14.7 ± 8.0
Lactate	3.3 ± 2.0
Creatinine	1.75 ± 1.7
Blood urea nitrogen (BUN)	27.3 ± 24
Total bilirubin	1.5 ± 4.0

The degree to which EPs agreed with how nurses interpreted US findings is detailed in Table 3. All EM-trained or board-certified physicians met hospital credentialing criteria and were deemed competent to perform and interpret POCUS. EPs on shift managing the septic patients were asked about their interpretation of the NP-POCUS images. EPs agreed with US interpretations performed by nursing staff in 100% of cases at hour 0, 97.3% of cases at hour 3, and 100% of cases at hour 6. Additionally, once EPs viewed the saved NP-POCUS images, they often changed fluid management and administered fluids per algorithm when compared to the EPs' initial patient assessment without ultrasound. EPs were allowed to perform their own POCUS assessment if desired; however, whether they did so was not tracked since the focus of the study was on the nurse-driven protocol to facilitate sepsis resuscitation. The code team was there to facilitate the transition of care for the first 6 hours and ensure the patient stayed on track with the completion of the sepsis bundle, especially while the EPs continued to manage other critical patients. NP-POCUS led to EPs changing fluid management in an average of 83.7% of the cases. In the event that reviewing nursing US images did not change management, EPs did report that NP-POCUS increased confidence in the current treatment plan in an average of 96.6% of the cases. Prior to viewing the NP-POCUS images, EPs underestimated total fluid tolerance in 37.5% of cases, overestimated total fluid tolerance in 26.0% of cases, and correctly estimated (within 500ml) total fluid tolerance in 36.5% of cases (Table 4). Over the course of resuscitation, IVCs became less collapsible, the number of cases with B-lines was essentially unchanged, and less fluid was recommended (Figure 2). Table 3 shows the physician survey of nurse ultrasound interpretation. 

**Table 3:** Physician survey of nurse ultrasound interpretation US: Ultrasound. Note: Nine subjects were not evaluated by physicians at hour zero and are excluded from this table. However, it was a small number that did not get hour zero data but did get hour three and six data points.

	Hour 0	Hour 3	Hour 6
Physician agrees with nurse US interpretation	95/95 (100%)	73/75 (97.3%)	61/61 (100%)
Ultrasound changes physician management	74/93 (79.6%)	64/74 (86.5%)	51/60 (85.0%)
Ultrasound increases physician confidence in current treatment plan	59/60 (98.3%)	45/48 (93.8%)	40/41 (97.6%)

**Table 4:** Accuracy of physician estimation of fluids tolerated in the first six hours of resuscitation before reviewing nursing-performed ultrasound.

Physician estimation of fluids accuracy	
Underestimated	39/104 (37.5%)
Overestimated	27/104 (26.0%)
Correctly estimated	38/104 (36.5%)

## Discussion

While extensive research has been conducted to understand the procedural benefits of training nurses to utilize US, this study sought to demonstrate that physician decision-making could be impacted through NP-POCUS. US technology has repeatedly proven to be a valuable diagnostic tool that allows physicians to provide better treatment to their patients [2-8,10-14]. Our results reflect this knowledge in that, before incorporating POCUS into patient assessment, physicians correctly estimated (within 500 mL) the six-hour fluid tolerance of septic patients in only 36.5% of cases. In cases where the use of US technology has expanded to medical professionals other than physicians, multiple studies have shown that training nurses to incorporate US into their procedures leads to improved quality of care and higher levels of patient satisfaction [11-20]. Despite the vast amount of knowledge regarding the success of POCUS training courses for nurses and how they improve nursing practices, there is a paucity of literature focusing on how NP-POCUS influences physician treatment decisions, specifically those impacting care plans for septic patients in the ED. 

This study was conducted with the primary objective of determining if NP-POCUS could influence treatment management decisions affecting septic patients in the ED. Our results confirm that properly trained nurses can effectively use POCUS to accurately assess the fluid status of septic patients, change the treatment plan originally envisioned by the physician, and increase physician confidence in the patient’s treatment plan.

POCUS was utilized to assess fluid status by measuring the collapsibility of the IVC and by scanning the lung fields for fluid overload, as indicated by the presence of B-lines. It was found that the IVC would become significantly less collapsible as more fluids were administered to a patient (approaching their maximum fluid tolerance), and B-lines did not significantly change with the patient’s fluid status. After nurses analyzed their POCUS scans and reported any decreased collapsibility of the IVC, there was a significant decrease in the amount of fluid ordered by the emergency physician. EPs in the study reported that NP-POCUS changed their envisioned treatment plan in the majority of cases (83%). It is also important to note that EP assessments of NP-POCUS images were in agreement with nursing staff assessments in 99% of cases. This finding is congruent with previous research stating that nursing staff can excel at utilizing US techniques and also suggests that proper training enables nurses to assess their scanned images with the same accuracy as physicians [19-20].

This study suggests that nurses trained in POCUS can help collaborate with EP in the management of septic patients. Further expansion in training for specialty nurses in the utilization of POCUS should be considered. Many medical professionals, especially those working in the ED, are responsible for making critical healthcare decisions in a stressful workplace environment. While individuals may develop different strategies to meet these challenges, research has shown how multitasking, workplace interruptions, and the sheer cognitive load forced onto emergency staff can be detrimental to patient care [26-29]. One way these effects could be diminished would be by incorporating more collaboration into the nursing and physician relationships between emergency medical professionals. Collaboration has been found to be an important factor in maintaining a positive nursing and physician relationship, and nurses generally view collaboration as being acknowledged by the physician and working together to devise a care plan for patients [30]. Research also shows that the nursing and physician relationship may indirectly impact patient outcomes by affecting nurse job satisfaction and their willingness to continue working at a particular institution [31]. In our study, the code team took on the primary role of serial fluid resuscitation in severe septic and septic shock patients in a timely manner, which was continued inpatient while waiting for admission orders. This partnership allowed the EPs to closely monitor these septic patients and allow them to deal with multiple other critical patients without falling behind on critical resuscitative efforts on their septic patients. 

With these facts in mind, POCUS training for nursing staff provides a unique way to foster interprofessional collaboration in the emergency department, improve the relationship between emergency care providers, and ultimately allow patients to receive more timely, higher-quality care.

Our study focused on the NP-POCUS fluid assessment for septic patients and facilitated care along with EPs. It was a qualitative study to see if EPs would agree with the RN’s interpretation of fluid responsiveness using POCUS. One of our limitations is that we did not look at patient-centered outcomes or mortality since this study was designed as a quality improvement project. We did not further evaluate patients with sepsis criteria based on their co-morbidities, such as ESRD or heart failure, since this subset of patients tends to get fluid resuscitation cautiously due to the risk of fluid overload. Other limitations include the lack of data from physicians performing their own POCUS exams, the convenience sampling method, the single-center design, or potential biases. Future studies should elaborate on the validity of NP-POCUS interpretations by comparing them to EP’s POCUS by a third-party expert to assess their accuracy. Other future projects should examine the long-term impact of NP-POCUS on patient outcomes, explore the cost-effectiveness of NP-POCUS, or test the validity of the study findings.

## Conclusions

Our study confirmed that it is both feasible and beneficial to train nurses in POCUS for fluid management to help treat septic patients in the ED. As shown in this study, emergency nurses can be trained using readily available online and in-person resources. The subset of specialized nurses, the code team, can facilitate septic patient care between EPs and continue the septic bundle until admission orders are placed to streamline critical treatment plans. Our results indicate that nursing-performed POCUS contributed to and, in some cases, altered patient management and that, in most cases, nursing US interpretations were consistent with EP interpretation. Our study also supports the potential of nursing-led POCUS for other clinical conditions, and we encourage further study of this potential. 
